# Theoretical Studies on Two-Photon Fluorescent Hg^2+^ Probes Based on the Coumarin-Rhodamine System

**DOI:** 10.3390/s17071672

**Published:** 2017-07-20

**Authors:** Yujin Zhang, Jiancai Leng

**Affiliations:** School of Science, Qilu University of Technology, Jinan 250353, China; zhangyujin312@163.com or zhangyujin@qlu.edu.cn

**Keywords:** fluorescent Hg^2+^ probe, time-dependent density functional theory, two-photon absorption

## Abstract

The development of fluorescent sensors for Hg^2+^ has attracted much attention due to the well-known adverse effects of mercury on biological health. In the present work, the optical properties of two newly-synthesized Hg^2+^ chemosensors based on the coumarin-rhodamine system (named Pro1 and Pro2) were systematically investigated using time-dependent density functional theory. It is shown that Pro1 and Pro2 are effective ratiometric fluorescent Hg^2+^ probes, which recognize Hg^2+^ by Förster resonance energy transfer and through bond energy transfer mechanisms, respectively. To further understand the mechanisms of the two probes, we have developed an approach to predict the energy transfer rate between the donor and acceptor. Using this approach, it can be inferred that Pro1 has a six times higher energy transfer rate than Pro2. Thus the influence of spacer group between the donor and acceptor on the sensing performance of the probe is demonstrated. Specifically, two-photon absorption properties of these two probes are calculated. We have found that both probes show significant two-photon responses in the near-infrared light region. However, only the maximum two-photon absorption cross section of Pro1 is greatly enhanced with the presence of Hg^2+^, indicating that Pro1 can act as a potential two-photon excited fluorescent probe for Hg^2+^. The theoretical investigations would be helpful to build a relationship between the structure and the optical properties of the probes, providing information on the design of efficient two-photon fluorescent sensors that can be used for biological imaging of Hg^2+^ in vivo.

## 1. Introduction

Mercury is a caustic and carcinogenic element with high cellular toxicity which can pass through biological membranes easily and cause serious damage to the neurological and endocrine systems [1,2,3], which makes the detection of mercuric ion (Hg^2+^) of great importance in the fields of biology, chemistry and medicine [4,5,6]. In the past few years, many analytical methods have been developed to monitor the concentration of Hg^2+^. Thereinto, the fluorescence microscopy technique has attracted much attention due to its high sensitivity and selectivity, a low cost [7,8]. The design of effective fluorescent probes consequently has become a focus of attention in fluorescence microscopy [9,10,11].

Until now, several recognition mechanisms have been employed in probe design [4,12,13]. At the very beginning, the intramolecular charge transfer (ICT) mechanism was employed. For instance, Srivastava et al. synthesized a fluorescent probe by bridging a benzhydryl moiety and a dansyl fluorophore through a piperazine unit to detect Hg^2+^ [14]. Razi et al. designed a fluorescence turn-on ratiometic probe for Hg^2+^ by bridging imidazole and benzothiazole moieties through a thiophene ring [15]. Even though much success has been achieved in the development of ICT-based fluorescent probes, the shortage of alternatives is evident. It is widely accepted that the detection using ICT-based fluorescent probes depends highly on the intensity of the single characteristic fluorescent band, which is usually affected by the environment and measurement conditions [16,17,18]. Other than the ICT mechanism, energy transfer-based mechanisms, including Förster resonance energy transfer (FRET) and through bond energy transfer (TBET) can eliminate the mentioned interferences by using the built-in correction provided by two-emission bands. For a probe based on FRET, the donor moiety and acceptor moiety are linked by a non-conjugated spacer. Consequently, the energy transfers from the donor to the acceptor rely on the spectral overlap between the donor emission and acceptor absorption [19,20]. On the other hand, a probe based on TBET is one whose donor is connected to the acceptor via an electronically conjugated linker. As a result, the energy transfer process occurs through the bond without the need for spectral overlap [21,22].

To date, considerable efforts have been devoted to developing energy transfer-based Hg^2+^ fluorescent probes [23,24,25]. Very recently, Gong et al. designed a coumarin-rhodamine TBET system (named hereafter as Pro1). They have demonstrated that Pro1 was particularly useful for ratiometric Hg^2+^ sensing and bioimaging applications [26]. Adopting the same donor (coumarin) and acceptor (rhodamine), Wang et al. reported another Hg^2+^ fluorescent probe in which a *m*-phenylenediamine spacer was used as the linker (named hereafter as Pro2) [27]. Although the experimental measurements show that both Pro1 and Pro2 are promising fluorescent probes for Hg^2+^, the underlying mechanism of the probes is insufficiently understood, in particular the role of the spacer in the sensing performance. Meanwhile, energy transfer rate is a very important parameter for evaluating the efficiency of a probe. However, there is no standard approach to evaluate the energy transfer efficiency in different experiments. Thus, comparison between probes’ energy transfer rates on the same theoretical level basis is needed. More importantly, in the experiments, Pro1 and Pro2 have been excited by short wavelength one-photon irradiation, which easily results in photobleaching, photodamage and interference from auto-fluorescence. An efficient method that can overcome the shortcomings of a one-photon fluorescent probe is to utilize a two-photon fluorescent probe [28,29,30,31], therefore, the potential of the probes Pro1 and Pro2 as two-photon fluorescent Hg^2+^ sensors should be investigated.

In this paper, theoretical studies on the optical properties, including one-photon absorption (OPA), one-photon emission (OPE) and two-photon absorption (TPA) of Pro1 and Pro2 in the absence and presence of Hg^2+^ were carried out. Special attention has been paid to the analysis of probes’ recognition mechanisms by illustrating the molecular orbital distributions involved in the photoabsorption and photoemission processes. Importantly, we report a feasible approach to predict the energy transfer rate of the probes. The present research should be helpful to understand the response mechanisms of these fluorescent chemosensors. Most of all, the role of spacer between the donor and acceptor of the probes is demonstrated, providing guidelines for the design of more efficient two-photon fluorescent probes.

## 2. Theoretical Method and Computational Details

### 2.1. Theoretical Method

The detailed theory has been reported in [32]. Here, only the main formulations are briefly discussed. One-photon absorption and emission strength between the states *i* and *j* can be described by the oscillator strength:

(1) $$\delta_{OPA(OPE)}=\frac{2\omega_{ij}}{3}\sum_{\alpha }^{x,y,z}|\langle i|\mu_{\alpha }|j\rangle {|}^{2},$$

where *ω_ij_* denotes the energy difference between the states *i* and *j*, *μ_α_* is the electric dipole moment operator.

The macroscopic TPA cross-section that can directly compare with the experimental value is defined as:

(2) $$\sigma_{TPA}=\frac{4\pi^{2}a_{0}{}^{5}\alpha \omega^{2}g(\omega )}{15c\Gamma }\delta_{TPA},$$

where *a*_0_ is the Bohr radius, *α* the fine structure constant, *c* is the speed of the light, and *ħω* is the incident photon energy. *g*(*ω*) provides the spectral line profile, and the lifetime broadening of the final state Γ is assumed to be a typical value of 0.1 eV [33]. *δ_TPA_* is the microscopic TPA cross-section which is given by the orientational averaging over the two-photon transition probability.

### 2.2. Computational Detail

The geometrical structures of all the studied molecules were fully optimized using the time-dependent density function theory (TD-DFT)/B3LYP exchange functional level with 6-31G(d,p) basis set. In order to verify the stability of the optimized structures, frequency calculations were performed and no imaginary frequency was obtained. On the basis of the optimized structures, OPA properties of the molecules are calculated using the functional of TD-DFT/BLYP and the 6-31G(d,p) basis set. It should be noted that other functionals, such as B3LYP, M06-2X, PBE0 and wB97X are also used for the numerical simulations. The results of BLYP functional agree however better with the experimental measurements. Thus, the BLYP functional was chosen for the property calculations. Meanwhile, simulations on the OPE properties were carried out by optimizing the first excited state of the compounds. All the above mentioned calculations are performed utilizing the Gaussian09 program (Gaussian Inc., Wallingford, CT, USA) [34]. Apart from the OPA and OPE properties, TPA properties of the studied molecules are also investigated based on the optimized ground state structures using the quadratic response theory implemented in the Dalton2013 package [35]. Considering that the experimental measurements are carried out in an aqueous environment, the effect of solvent is taken into account within the polarizable continuum model (PCM) in all calculations.

## 3. Result and Discussion

### 3.1. Molecular Geometry

**Figure 1:**
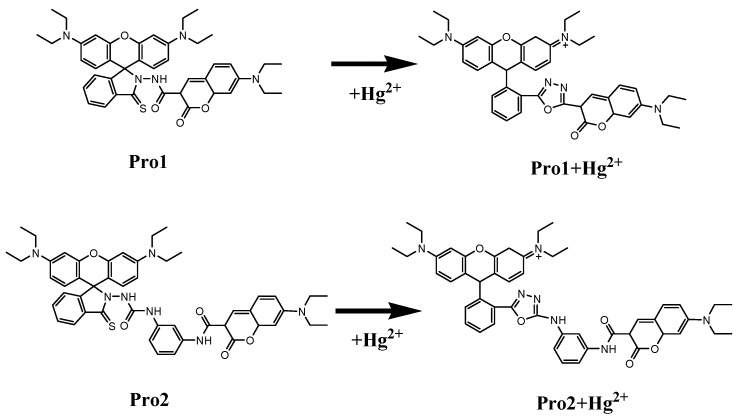
Molecular structures of Pro1, Pro1 + Hg^2+^, Pro2 and Pro2 + Hg^2+^.

Figure 1 shows the structures of the studied molecules. Both Pro1 and Pro2 employ rhodamine moiety as the acceptor, and a coumarin moiety with excellent biocompatibility as the donor. In Pro1, the acceptor and donor are connected directly, whereas in Pro2, a rigid *m*-phenylenediamine was selected as the spacer group. 

**Figure 2:**
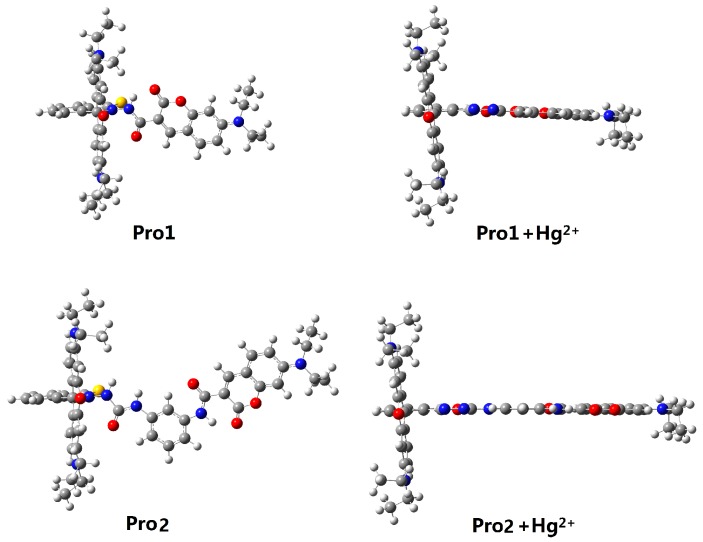
Optimized ground state geometries of Pro1, Pro1 + Hg^2+^, Pro2 and Pro2 + Hg^2+^ with PCM simulating the dielectric of water.

In the absence of Hg^2+^, the rhodamine moiety adopts a closed, non-fluorescent spirolactam form, as shown in Pro1 and Pro2. In the presence of Hg^2+^, a Hg^2+^-promoted reaction will induce opening of the rhodamine moiety as shown in Pro1 + Hg^2+^ and Pro2 + Hg^2+^. Optimized ground state molecular geometries are given in Figure 2. In all molecular structures, the donors are planar, and in the acceptors both for closed-ring form and open-ring form, the benzene and the xanthene are vertical. Notably, there is a large torsion between the donor and acceptor of the molecules. This non-coplanar characteristic of the probes prevents the molecule from behaving as a conjugated dye, so that the energy can transfer between the two parts.

### 3.2. One-Photon Absorption

**Figure 3:**
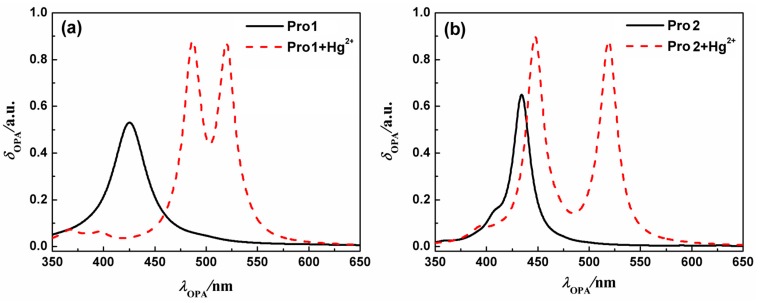
The OPA spectra of (**a**) Pro1 and Pro1 + Hg^2+^, (**b**) Pro2 and Pro2 + Hg^2+^ with PCM simulating the dielectric of water.

On the basis of the optimized geometries, OPA properties of the studied molecules in H_2_O were calculated. Figure 3 shows the absorption spectra of Pro1, Pro1 + Hg^2+^, Pro2 and Pro2 + Hg^2+^. The details of the OPA peaks, including the one-photon absorption energy, the corresponding wavelength, the oscillator strength and the transition nature are presented in Table 1. It can be seen from Figure 3 and Table 1 that Pro1 and Pro2 have one absorption peak at 425 nm and 434 nm, which agrees well with the experimental data of 420 nm and 440 nm, respectively. When reacts with Hg^2+^, both Pro1 + Hg^2+^ and Pro2 + Hg^2+^ simultaneously show two absorption peaks, i.e., 486 nm and 520 nm for Pro1 + Hg^2+^, 447 nm and 521 nm for Pro2 + Hg^2+^. In comparison with the maximum OPA for the free probes, absorption peaks for the compounds upon combing with Hg^2+^ show redshifts. Predictably, the absorption peak at about 520 nm for Pro1 + Hg^2+^ and Pro2 + Hg^2+^ is dominated by the open-ring rhodamine. The shift of the absorption peak in the short wavelength range is much larger for Pro1 + Hg^2+^ than Pro2 + Hg^2+^, which is consistent with the trend if the experimental measurements.

**Figure 4:**
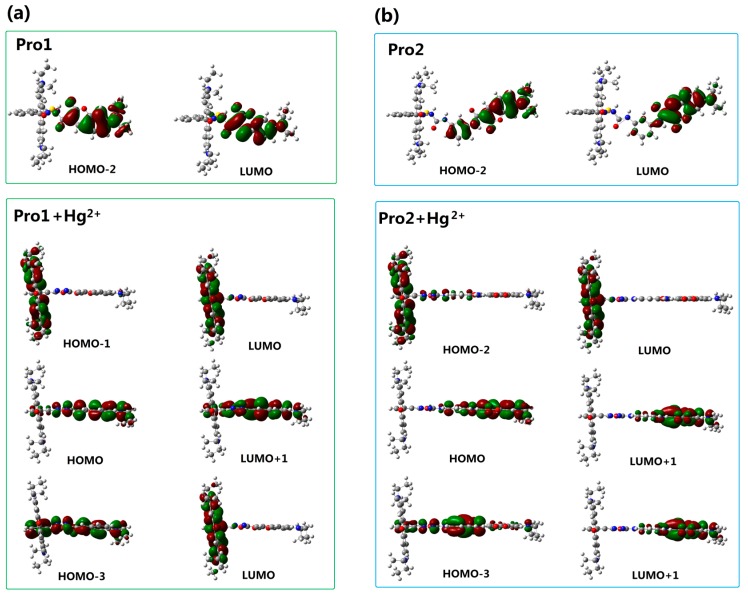
Molecular orbitals involved in the transition of the OPA peaks for (**a**) Pro1 and Pro1 + Hg^2+^; (**b**) Pro2 and Pro2 + Hg^2+^ with PCM simulating the dielectric of water.

To better explain the spectral phenomena, the molecular orbitals contribute to the transitions corresponding to each OPA peak (see Table 1) are shown in Figure 4. It can be observed from Figure 4a that the maximum absorption of Pro1, which originates from the HOMO-2 to the LUMO (HOMO and LUMO represent the highest occupied molecular orbital and the lowest unoccupied molecular orbital, respectively) transition, is distributed on the donor moiety. In the presence of Hg^2+^, the long wavelength absorption of Pro1 + Hg^2+^ at 520 nm is attributed to the HOMO-1 to LUMO transition and localized on the acceptor part. The short wavelength absorption of Pro1 + Hg^2+^ at 486 nm results from both the HOMO to LUMO + 1 transition and the HOMO-3 to LUMO transition. It should be noted that the HOMO and LUMO + 1 of Pro1 + Hg^2+^ are localized on the donor part, whereas the HOMO-3 and LUMO are localized on the donor and acceptor moiety, respectively. Thus the short wavelength absorption peak for Pro1 + Hg^2+^ at 486 nm relates to the charge transfer process between the donor and the acceptor, which leads to a larger redshift compared with the absorption peak of Pro1. From Figure 4b, it can be seen that similar change trends are shown for the absorption of Pro2 and the long wavelength absorption of Pro2 + Hg^2+^. However, the short wavelength absorption of Pro2 + Hg^2+^ at 447 nm is contributed by the donor itself, revealing that there is no electronic interaction between the donor and acceptor for Pro2 + Hg^2+^ upon excitation due to the *m*-phenylenediamine spacer. Analyses on the OPA of the molecules suggest that the donor and acceptor of Pro1 + Hg^2+^ and Pro2 + Hg^2+^ can be individually excited at their characteristic absorption peaks, which is conducive to the energy transfer process.

### 3.3. One-Photon Emission

**Figure 5:**
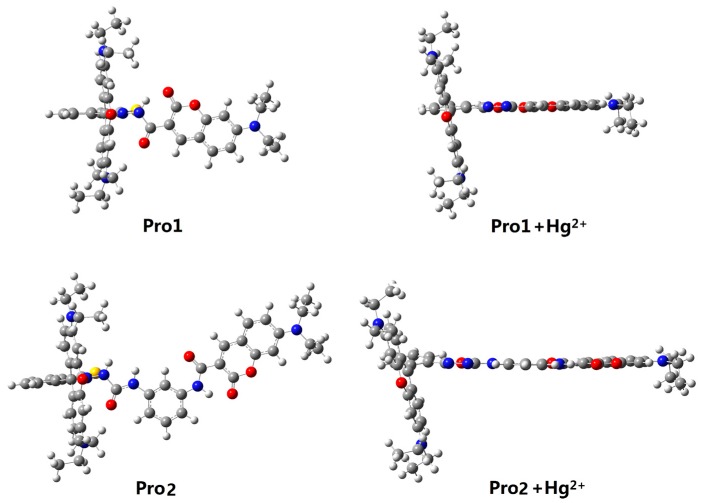
Optimized first excited state geometries of Pro1, Pro1 + Hg^2+^, Pro2 and Pro2 + Hg^2+^ with PCM simulating the dielectric of water.

For a ratiometric fluorescent probe, great changes on the fluorescent signal should be exhibited when it reacts with the target analyte. Calculations on the OPE properties of the studied molecules in H_2_O are performed on basis of the optimized first excited state geometries. The optimized geometries of Pro1, Pro1 + Hg^2+^, Pro2 and Pro2 + Hg^2+^ in the first excited state are shown in Figure 5. Compared with the ground state geometries, the first excited state geometries show little change.

**Figure 6:**
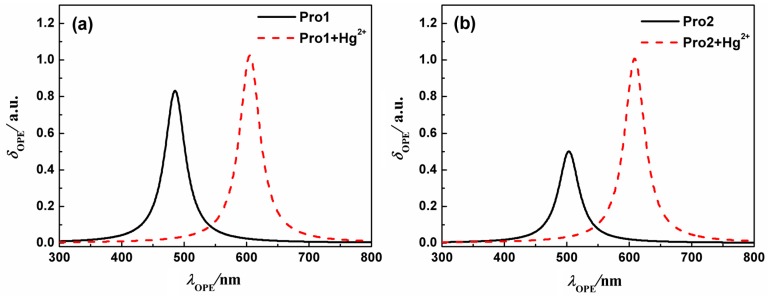
The OPE spectra of (**a**) Pro1 and Pro1 + Hg^2+^, (**b**) Pro2 and Pro2 + Hg^2+^ with PCM simulating the dielectric of water.

At the same time, the fluorescence spectra of the molecules in H_2_O are presented in Figure 6, and the detailed OPE energy, the corresponding wavelength, the fluorescent intensity and the transition nature are listed in Table 2. Values in Table 2 show that upon the addition of Hg^2+^, the fluorescent peaks of Pro1 and Pro2 locating at 485 nm and 503 nm are greatly redshifted to 605 nm and 608 nm, showing 120 nm and 105 nm shifts, respectively. The calculated results agree well with the experimental data in trend, despite the quantitative discrepancy which can be attributed to the vibrational contribution, and the short-range interaction that have not been considered in the calculations, etc. By analyzing the OPE properties of the molecules, one can predict that these probes can act as excellent ratiometric chemosensors for Hg^2+^, and Pro1 is preferable due to its more apparent changes in the fluorescent wavelength. Considering the difference of the molecular structure, the influence of the connection between the donor and acceptor on the molecular fluorescence is revealed.

**Figure 7:**
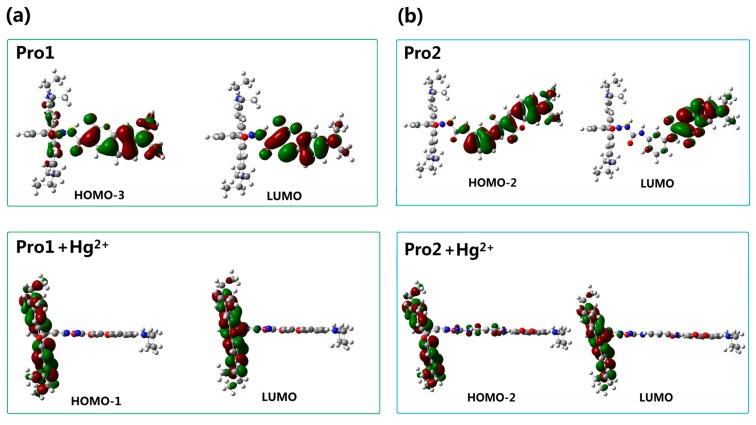
Molecular orbitals involved in the transition of the OPE peaks for (**a**) Pro1 and Pro1 + Hg^2+^; (**b**) Pro2 and Pro2 + Hg^2+^ with PCM simulating the dielectric of water.

In order to understand the fluorescence spectra phenomenon more clearly, the frontier molecular orbitals related to the main transitions in the emission process of the molecules are drawn and illustrated in Figure 7. It can be seen that the transitions of the emission for Pro1 and Pro2 are contributed by the orbitals localized on the donor moiety, whereas, the emission of Pro1 + Hg^2+^ and Pro2 + Hg^2+^ are dominated by the orbitals distributing on the acceptor part, resulting in the appearance of a characteristic emission of the acceptor.

For the probes before and after reacting with Hg^2+^, the frontier molecular orbitals involved in the fluorescent emission process are entirely localized on the donor or acceptor moiety. This makes the generation of separate characteristic emission peaks possible and is in favor of the recognition of Hg^2+^.

### 3.4. Recognition Mechanism and Energy Transfer Rate

As reported by Gong and Wang, the recognition of Pro1 and Pro2 for Hg^2+^ pertains to the TBET and FRET mechanism, respectively. However, theoretical analyses on this issue have not been carried out yet. Thus, to further rationalize the recognition mechanisms of Pro1 and Pro2, the orbital distribution diagrams included in the absorptive and emissive processes of the molecules are used to investigate the responsive process of a molecule upon optical excitation. Careful inspection of the absorption and emission of Pro1 + Hg^2+^ (see Figure 4a and Figure 7a) reveals that when excited by the incident light with the wavelength of 486 nm, Pro1 + Hg^2+^ can be excited to a higher excited state, which corresponds to an electronic transition mainly localized on the donor part, whereas, the emission of the molecule originating from the decay from the first excited state to the ground state is distributed on the acceptor moiety with a fluorescence wavelength of 605 nm. To summarize, the fluorescence of the molecular acceptor can be stimulated by exciting the donor moiety, herein a non-radiative energy transfer process occurs between the molecular donor and acceptor. Similarly, the energy transfer process occurs for Pro2 + Hg^2+^ as shown in Figure 4b and Figure 7b. Namely, the emission from the acceptor moiety of Pro2 + Hg^2+^ could be obtained by exciting the donor unit. Further taking the connection and the spectral overlap between the donor and acceptor into consideration, the recognition mechanisms of Pro1 and Pro2 are confirmed as TBET and FRET, respectively.

For an energy transfer-based system, the energy transfer rate between the donor and acceptor is an important parameter to evaluate the efficiency of the probe. According to the Fermi’s golden rule, the energy transfer rate from the donor to the acceptor, which represents the probability of the energy transfer per unit time, can be described by [36]:

(3) $$K_{DA}=\sum_{i,j}\frac{2\pi }{\hbar }{\int \left|W_{D_{i}A_{j}}\right|}^{2}\delta_{D_{i}}^{2}\delta_{A_{j}}^{2}\frac{\Gamma^{2}}{\pi^{2}\left[{\left(\varepsilon -\varepsilon_{D_{i}}\right)}^{2}+\Gamma^{2}\right]\left[{\left(\varepsilon -\varepsilon_{A_{j}}\right)}^{2}+\Gamma^{2}\right]}g(\varepsilon )d\varepsilon ,$$

where *ε_Di_*(*ε_Aj_*) is the transition energy of the donor(acceptor), *δ_Di_*(*δ_Aj_*) is the corresponding strength. *g*(*ε*) represents the energy distribution of the incident laser pulse which is assumed to be a Gaussian function. *W_DiAj_* denotes the electronic dipole-dipole interaction matrix element. Under the dipole approximation, *W_DiAj_* can be defined as:

(4) $$W_{D_{i}A_{j}}=\frac{\mu_{m}\cdot \mu_{n}}{|R_{mn}{|}^{3}}-3\frac{\left(\mu_{m}\cdot R_{mn})(\mu_{n}\cdot R_{mn}\right)}{|R_{mn}{|}^{5}},$$

here ***μ****_m_*(***μ****_n_*) is the transition dipole moment of the molecular fragment *m*(*n*), ***R****_mn_* is the distance vector from *m* to *n*.

**Figure 8:**
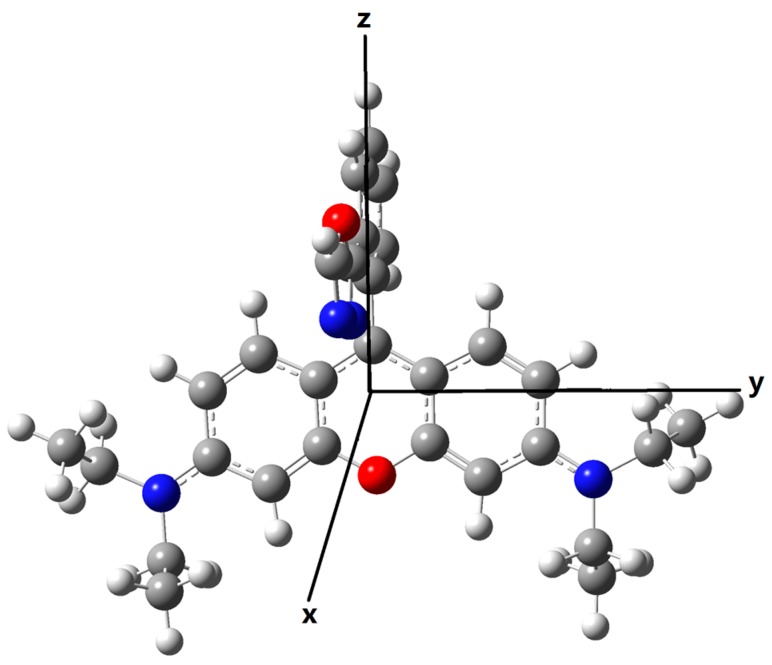
Schematic of coordinate direction.

Ideally, the transition dipole moments of donor emission and the acceptor absorption should have large vectors in the same direction, which is beneficial to the long-range dipole-dipole interaction. Here as shown in Figure 8, the *X*-axis is set to be perpendicular to the plane of the xanthene in rhodamine, the *Y*-axis on the long axis direction of the xanthene, and the *Z*-axis on the short axis of the xanthene. After fixing the coordinate direction, the transition wavelength, the corresponding strength and dipole moments for both the emission of the donors and the absorption of the acceptor (open-ring rhodamine) are calculated and the results are listed in Table 3. One can see that the emission wavelength of the donor for Pro2 is much closer to the absorption wavelength of the acceptor part than that for Pro1. Thus, spectral overlap between the donor emission and the acceptor absorption for Pro2 + Hg^2+^ is large while that of Pro1 + Hg^2+^ is small, proving the recognition mechanisms of Pro2 + Hg^2+^ to be FRET. In addition, there are large transition dipole moment vectors in the *Z* direction for the donors of Pro1 and Pro2, whereas the maximum transition dipole moment of the acceptor part is on the *Y* direction.

According to Equation (4), the energy transfer rate from the donor to the acceptor depends crucially on the distance between the two parts. Table 4 lists the distance vectors between the donor and the acceptor for Pro1 + Hg^2+^ and Pro2 + Hg^2+^. Obviously, the *m*-phenylenediamine spacer in Pro2 + Hg^2+^ leads to a long distance between the molecular donor and acceptor. As a result, the energy transfer rate of Pro1 + Hg^2+^ is six times larger than that of Pro2 + Hg^2+^, suggesting Pro1 + Hg^2+^ to be a more promising energy transfer-based probe. It can be concluded from these results that the spacer group between the donor and acceptor plays an important role on the efficiency of the energy transfer-based fluorescent probes.

### 3.5. Two-Photon Absorption

The analyses on the one-photon absorption and emission of the molecules demonstrate that Pro1 and Pro2 can efficiently identify Hg^2+^ through the energy transfer process. This inspired us to further investigate the utility of the probes as two-photon fluorescent chemosensors. The TPA properties of the molecules, including the two-photon absorption energy, the corresponding TPA wavelength and the TPA cross-section are listed in Table 5. In the case of free probes, both Pro1 and Pro2 have one TPA peak at about 750 nm. The maximum TPA cross section of Pro2 is 573.12 GM, which is six times larger than that of Pro1. One can expect the different TPA performances of Pro1 and Pro2 are due to their different spacers. Herein, we analyze the TPA cross-sections of the two probes by a two-level model with the ground state and the maximum charge transfer state [37] according to the transition natures of the probes as shown in Table 1. In this model, the peak TPA cross-section is directly proportional to the square of the transition dipole moment *μ*_01_, while inversely proportional to the square of the energy gap *Ε*_01_, namely, *σ_TPA_* ∝ *μ*_01_^2^/*Ε*_01_^2^. The numerical results are given in Table 6. From Table 6, one can see that Pro1 has a smaller transition dipole moment and larger excitation energy compared with Pro2, thus the TPA cross section of Pro1 is much smaller than that of Pro2.

**Figure 9:**
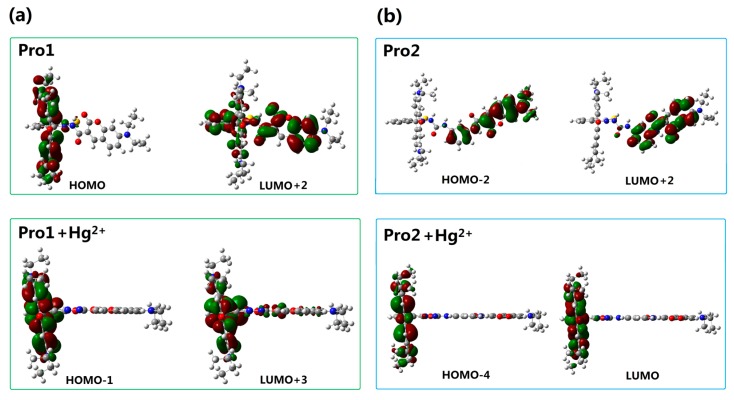
Molecular orbitals involved in the transition of the TPA peaks for (**a**) Pro1 and Pro1 + Hg^2+^, (**b**) Pro2 and Pro2 + Hg^2+^ with PCM simulating the dielectric of water.

With the addition of Hg^2+^, it can be seen that the TPA peak positions of Pro1 + Hg^2+^ and Pro2 + Hg^2+^ are blue shifted and red shifted, respectively. Importantly, the maximum TPA cross sections of Pro1 + Hg^2+^ is largely increased to 1059.10 GM, whereas that of Pro2 + Hg^2+^ is decreased to 275.96 GM. This demonstrates that the spacer group between the donor and acceptor of the probe induces a variation in its TPA property. Considering the molecules have no symmetry, the transition probability between two molecular orbitals is closely related to the overlap of these molecular orbitals [38]. Thus, molecular mainly orbitals involved in the TPA process of the studied molecules are drawn in Figure 9. In comparison with Pro1, the orbitals of Pro1 + Hg^2+^ contributing to the TPA process locate at the same moiety and possess similar distribution. Nevertheless for Pro2 + Hg^2+^, the overlapped parts take an opposite distribution, namely, color distribution of the overlapped part is opposite, resulting in destructive interference of the corresponding wavefunctions. As a consequence, the TPA cross-section of Pro1 + Hg^2+^ is enhanced and that of Pro2 + Hg^2+^ is reduced compared with Pro1 and Pro2, respectively.

Undoubtedly, an excellent two-photon fluorescent probe should have a significant enhancement in the TPA cross-section when identifying the target. Thus, Pro1 is predicted to be a better two-photon fluorescent probe. For Pro1, using a 777 nm laser as the two-photon excitation light, the probe is excited and the fluorescence emitted from the donor can be observed because no energy transfer process occurs. When Hg^2+^ is present and excited by two-photon irradiation, a TBET process occurs in Pro1 + Hg^2+^, and the fluorescence of the acceptor can be observed. In a word, Pro1 can be used as an active TBET-based two-photon fluorescent Hg^2+^ probe for live cell imaging.

## 4. Conclusions

In this work, the photoabsorption and photoemission properties, recognition mechanisms and energy transfer rates of two newly-synthesized fluorescent Hg^2+^ chemosensors were studied by theoretical calculations. The results demonstrate that obvious changes in the absorption and fluorescence signal of the probes are observed upon reaction with Hg^2+^, which is conductive to the recognition of this cation. The analyses on the energy transfer rate illustrate that Pro1 has a higher efficiency, therefore, Pro1 is proved to be a more effective energy transfer-based ratiometric fluorescent probe for detecting Hg^2+^. Then the effect of spacer group between the donor and acceptor on the sensing performance and efficiency of the probe is discussed. In particular, the probes show a significant two-photon response in the near-infrared light region, and the largest TPA cross-section of Pro1 is greatly enhanced with the presence of Hg^2+^. As a result, it is deduced that Pro1 can act as a potential two-photon excited TBET-based ratiometric fluorescent Hg^2+^ probe. The theoretical investigations have explained the experimental results and revealed the underlying response mechanism of the probes. Meanwhile, a new strategy to predict the energy transfer rate of the energy transfer-based chemosensor on basis of the molecular structure is proposed. The results are intended to give the structure-property relationships for these probes, providing useful knowledge for designing more efficient two-photon fluorescent sensors geared toward biological applications.

## Figures and Tables

**Table 1:** The one-photon absorption energy *E_OPA_* (eV), the corresponding wavelength *λ_OPA_* (nm), the oscillator strength *δ_OPA_* (a.u.), and the transition nature of the OPA peaks for Pro1, Pro1 + Hg^2+^, Pro2 and Pro2 + Hg^2+^ with PCM simulating the dielectric of water.

Molecule	*E_OPA_*	*λ_OPA_*	*λ_OPA_*	Transition Nature	$\lambda_{\mathrm{OPA}}^{\mathrm{Exp}}$
Pro1	2.92	425	0.53	HOMO-2 → LUMO 70%	420 ^a^
Pro1 + Hg^2+^	2.38	520	0.88	HOMO-1 → LUMO 92%	567 ^a^
	2.55	486	0.89	HOMO → LUMO + 1 60%, HOMO-3 → LUMO 12%	450 ^a^
Pro2	2.86	434	0.64	HOMO-2 → LUMO 98%	440 ^b^
Pro2 + Hg^2+^	2.37	521	0.87	HOMO-2 → LUMO 94%	568 ^b^
	2.77	447	0.90	HOMO → LUMO + 1 55%, HOMO-3 → LUMO + 1 30%	440 ^b^

^a^ Measured in 50:50 (*v*/*v*) THF-H_2_O [26] and ^b^ measured in 50:50 (*v*/*v*) EtOH-H_2_O [27].

**Table 2:** The emission energy *E_OPE_* (eV), the corresponding wavelength *λ_OPE_* (nm), the fluorescent intensity *δ_OPE_* (a.u.) and the transition nature of the OPE peaks for Pro1, Pro1 + Hg^2+^, Pro2 and Pro2 + Hg^2+^ with PCM simulating the dielectric of water.

Molecule	*E_OPE_*	*λ_OPE_*	*δ_OPE_*	Transition Nature	$\lambda_{\mathrm{OPA}}^{\mathrm{Exp}}$
Pro1	2.55	485	0.83	HOMO-3 → LUMO 85%	470 ^a^
Pro1 + Hg^2+^	2.05	605	1.02	HOMO-1 → LUMO 98%	580 ^a^
Pro2	2.46	503	0.50	HOMO-2 → LUMO 92%	478 ^b^
Pro2 + Hg^2+^	2.04	608	1.01	HOMO-2 → LUMO 94%	587 ^b^

^a^ Measured in 50:50 (*v*/*v*) THF-H_2_O [26] and ^b^ measured in 50:50 (*v*/*v*) EtOH-H_2_O [27].

**Table 3:** The transition wavelength *λ* (nm), transition strength *δ* (a.u.) and the corresponding transition electric dipole moments *μ_x,y,z_* (a.u.) of donor emission and acceptor absorption for Pro1 + Hg^2+^ and Pro2 + Hg^2+^ with PCM simulating the dielectric of water.

Molecule	*λ*	*δ*	Transition Electric Dipole Moment		
			*μ_x_*	*μ_y_*	*μ_z_*
Pro1-Donor-emission	396	0.69	−0.18	−0.20	−2.98
Pro2-Donor-emission	441	0.98	−0.06	−0.19	3.78
Acceptor-absorption	474	1.00	−0.14	−3.94	−0.24

**Table 4:** The distance vector *R_x,y,z_* (Å), total distance *R_DA_* (Å) and energy transfer rate *K_DA_* (10^6^) between the donor and acceptor of Pro1 + Hg^2+^ and Pro2 + Hg^2+^ with PCM simulating the dielectric of water.

Molecule	*R_x_*	*R_y_*	*R_z_*	*R_DA_*	*K_DA_*
Pro1 + Hg^2+^	8.28	0.55	2.07	8.55	3.7
Pro2 + Hg^2+^	15.74	0.31	1.98	15.87	0.6

**Table 5:** The two-photon absorption energy *E_TPA_* (eV), the corresponding TPA wavelength *λ_TPA_* (nm) and the TPA cross section *σ_TPA_* (GM = 10^−5^ cm^4^·s/photon) of the lowest nine excited states for Pro1, Pro1 + Hg^2+^, Pro2 and Pro2 + Hg^2+^ with PCM simulating the dielectric of water.

Molecule	*E_TPA_*	*λ_TPA_*	*σ_TPA_*	Molecule	*E_TPA_*	*λ_TPA_*	*σ_TPA_*
Pro1	2.72	909	3.14	Pro1 + Hg^2+^	2.38	1040	21.35
	2.87	861	3.45		2.55	972	24.34
	2.92	850	2.47		2.87	861	135.22
	2.98	829	1.99		3.08	802	264.19
	3.18	777	94.85		3.19	775	11.26
	3.33	742	0.09		3.24	763	33.23
	3.42	723	2.24		3.6	686	1059.1
	3.56	694	11.7		3.67	673	233.94
	3.63	681	0.42		3.79	652	382.14
Pro2	2.86	868	0.03	Pro2 + Hg^2+^	2.37	1042	1.83
	2.88	858	4.71		2.45	1009	0.47
	2.92	846	1.87		2.53	977	23.45
	3	824	0.41		2.77	894	0.36
	3.06	808	8.44		3.09	800	263.31
	3.18	777	1.17		3.28	754	275.96
	3.34	740	573.12		3.49	708	4.71
	3.5	706	0.02		3.54	698	40.68
	3.55	696	1.55		3.58	690	85.94

**Table 6:** The transition dipole moment *μ* (Debye) and excitation energy *E*_01_ (eV) between the states in two-level model for Pro1 and Pro2.

Molecule	*μ_x_*	*μ_y_*	*μ_z_*	*μ_tot_*	*E*_01_
Pro1	0.50	0.03	2.66	2.71	2.92
Pro2	0.51	0.83	2.85	3.02	2.86
